# Identifying a Critical Blind Spot: How Commercial AI (CAD) Systems Fail to Detect Faint Ground-Glass Opacities at −730 HU on Low-Dose CT

**DOI:** 10.3390/diagnostics16071014

**Published:** 2026-03-27

**Authors:** Shan Liang, Jia Wang, Wentao Fu, Yali Wang

**Affiliations:** Department of Radiology, Hebei Yiling Hospital, Shijiazhuang 050091, China

**Keywords:** computer-aided detection (CAD), pulmonary nodules, ground-glass opacity (GGO), false negative, CT value, quantitative analysis

## Abstract

**Objective:** The integration of artificial intelligence (AI) into computer-aided detection (CAD) is a major innovation in lung cancer diagnosis. However, its reliability in detecting the earliest radiographic sign—faint ground-glass opacities (GGOs) indicating pre-invasive adenocarcinoma—remains a critical, unquantified gap. This study aimed to perform a rigorous failure analysis to define the specific conditions under which commercial AI/CAD systems fail in a low-dose CT (LDCT) screening setting. **Methods:** In this retrospective diagnostic accuracy study, a primary cohort of 100 patients and an external validation cohort of 50 patients with moderate/low-risk nodules on LDCT were included. An expert reference standard was established by a consensus panel of three thoracic radiologists. Two independent, commercially deployed AI/CAD systems from different vendors (Vendor A & Vendor B) processed all cases. Nodules confirmed by experts but missed by AI were analyzed. Their morphology was categorized, and their mean CT attenuation (HU) was measured via manual region-of-interest placement. **Results:** The AI systems demonstrated significant and comparable false negative rates in the combined cohort: 12.7% for Vendor A and 14.7% for Vendor B. The vast majority of missed nodules were GGOs (92.3% and 78.6%, respectively, in the primary cohort). Crucially, quantitative analysis revealed a consistent density threshold for AI failure: the mean CT value of missed GGOs was −737 ± 51.50 HU for Vendor A and −727 ± 70.07 HU for Vendor B. This algorithmic blind spot was fully corroborated by the external validation cohort (−741 ± 48.2 HU and −733 ± 62.5 HU, respectively). Anatomical complexity (juxta-pleural/endobronchial location) was a secondary failure factor. **Conclusions:** This study identifies a quantifiable “−730 HU blind spot” as a common limitation of current commercial AI/CAD systems in diagnosing early lung adenocarcinoma. This finding represents a pivotal advancement in understanding AI’s role in diagnostics: it is not infallible. To innovate and safeguard screening efficacy, radiologists must adopt a human–AI collaborative model with mandated manual verification targeting low-attenuation opacities, ensuring this diagnostic innovation fulfills its promise while mitigating the risks of overdiagnosis.

## 1. Introduction

Lung cancer remains the leading cause of cancer-related mortality globally, necessitating effective early screening strategies [1]. The advent of Low-Dose Computed Tomography (LDCT) has revolutionized the detection of early-stage pulmonary lesions, with high-resolution, low-radiation CT enabling the identification of millimetric nodules that may represent pre-invasive adenocarcinoma [2]. Among these, ground-glass opacities (GGOs) and sub-solid nodules are of particular clinical concern. Although they often demonstrate indolent growth, persistent GGOs have a high probability of being malignant (e.g., minimally invasive adenocarcinoma) compared to solid nodules [3,4]. Early identification and resection of such lesions, often via sublobar resection, can lead to 5-year survival rates approaching 100%, frequently sparing patients from adjuvant chemotherapy or radiation [5].

To manage the immense workload generated by LDCT screening and to mitigate perceptual errors associated with radiologist fatigue, computer-aided detection (CAD) systems have become integral to modern radiology workflows [5,6]. Contemporary CAD systems, evolving from rule-based algorithms to advanced deep learning architectures, serve as a “second pair of eyes,” significantly improving nodule detection sensitivity [7,8,9]. However, despite these technological strides, “missed diagnosis” or false negative detection remains a critical challenge. The heterogeneity of pulmonary nodules—varying in size, density, morphology, and anatomical context—poses significant difficulties for automated algorithms [7].

The recent literature suggests that while CAD systems achieve high sensitivity for solid nodules, their performance significantly degrades for lesions with low contrast-to-noise ratios (CNRs), such as faint pure ground-glass opacities (GGOs), which are challenging to detect due to their subtle attenuation difference from surrounding lung parenchyma [10]. This limitation extends to subsolid nodules, where CAD sensitivity is notably lower for pure GGOs compared to part-solid nodules [11]. Understanding the specific failure modes of these systems is crucial for radiologists to effectively utilize AI tools. If a radiologist is unaware of the specific “blind spots” of a CAD system (e.g., its reduced sensitivity for low-attenuation or subsolid lesions), they may over-rely on a negative result, leading to missed diagnoses of potentially curable cancers.

However, it is equally imperative to contextualize the clinical significance of these faint, AI-missed lesions. What is the actual risk that minute GGOs missed by AI will progress into life-threatening carcinomas? In the context of population screening, detecting every single pre-malignant lesion is not unequivocally beneficial. Striking a delicate balance to avoid unnecessary surgical interventions—mitigating overdiagnosis and overtreatment—remains a central tenet of thoracic medicine. As outlined by the 2017 Fleischner Society Guidelines [12] and demonstrated in landmark prospective studies by Kakinuma et al. (2016) [13], GGOs measuring 5 mm or less frequently exhibit negligible volumetric growth, and patients harboring them may complete their natural lifespan without disease progression. Therefore, CAD blind spots are not analyzed solely to demand 100% algorithmic sensitivity, but to understand which specific sub-visual thresholds algorithms operate under, thereby empowering clinicians to make informed, balanced surveillance decisions.

Therefore, this study aims to move beyond simple performance metrics and conduct a failure analysis of two commercially available CAD systems. By quantitatively analyzing the nodules unrecognized by these systems—specifically focusing on CT attenuation values and morphological characteristics—we seek to define the “danger zones” for CAD failure. This research provides evidence-based recommendations for manual verification, ensuring that moderate and low-risk nodules, particularly low-density GGOs, are not overlooked in the era of AI-assisted screening.

## 2. Materials and Methods

This study was conducted in accordance with the Declaration of Helsinki and was approved by the ethics committee of our hospital. Patient data were anonymized to protect privacy. The reporting of this study follows the STARD (Standards for Reporting of Diagnostic Accuracy) guidelines [14].

### 2.1. Study Population and Eligibility Criteria

We retrospectively collected data from a primary cohort of 100 patients who underwent chest LDCT screening at our institution between January 2022 and December 2023. The cohort consisted of 60 males and 40 females, with an age range of 17–80 years (mean age 47.9 ± 11.30 years). To validate the reliability of our findings and address potential single-center bias, an external validation cohort comprising 50 patients (31 males, 19 females; mean age, 49.2 ± 10.8 years) was retrospectively collected from a secondary medical center under identical inclusion protocols.

**Inclusion Criteria:** (1) Availability of complete clinical data and high-quality LDCT images; (2) presence of at least one moderate or low-risk pulmonary nodule identified during initial screening. According to the Chinese Guidelines for the Classification, Diagnosis, and Treatment of Lung Nodules (2016) [15], moderate-risk nodules are defined as solid nodules (5–15 mm) without overt malignant signs, sub-solid nodules (<8 mm), or pure GGOs (>5 mm). Nodules smaller than these thresholds were classified as low-risk. The dataset included solid, sub-solid (part-solid), and pure GGO nodules.

**Exclusion Criteria:** Patients were meticulously excluded based on specific clinical and technical rationales. First, cases with significant image artifacts (e.g., severe respiratory motion blurring or streak artifacts from metallic implants) were excluded because these disruptions fundamentally corrupt the local density (HU) analysis and automated segmentation processes. Second, patients with concomitant diffuse lung diseases (such as severe interstitial pneumonia or massive lobar consolidation) and those with large pleural effusions were omitted, as these background pathologies drastically alter the baseline lung parenchyma attenuation, rendering accurate contrast-to-noise evaluation of nodules impossible. Finally, individuals with a known history of prior lung cancer, prior thoracic surgery, pregnancy, or lactation were excluded to ensure a standardized, naive screening population without altered post-surgical anatomy or radiation contraindications.

### 2.2. Image Acquisition

CT scans were performed using two multidetector MSCT scanners: a uCT 510 scanner (Shanghai United Imaging Healthcare Co., Ltd., Shanghai, China) and a LightSpeed VCT scanner (GE Medical Systems, LLC, Waukesha, WI, USA). Patients were trained in breath-holding techniques prior to scanning. The scan range extended from the lung apex to the lung base. Scanning parameters were: slice thickness of 5 mm, interval of 5 mm, pitch of 1.375, 512 × 512 matrix, and tube voltage of 100 kV, with automatic tube current modulation. Images were reconstructed with slice thicknesses of 1.3 mm (Vendor A) and 1.2 mm (Vendor B) using standard and lung kernels. Lung window settings were width 1500 HU/level −600 HU; mediastinal window settings were width 400 HU/level 40 HU.

### 2.3. Reference Standard (Ground Truth)

To establish the ground truth, three senior radiologists (with >10 years of experience in thoracic imaging) independently reviewed all images on a PACS workstation. They utilized manual electronic calipers to measure nodule dimensions. In cases of multiple nodules, the most clinically significant nodule (based on size or morphology) was selected for analysis. Discrepancies in detection or measurement were resolved through consensus discussion to establish the final reference dataset. A total of 150 clinically significant nodules (100 from the primary cohort, 50 from the validation cohort) formed the reference standard.

### 2.4. CAD Systems and Nodule Detection

Two separate anonymized commercial CAD systems were evaluated:

Vendor A: Integrated with the native CT platform (software: uWS-CT, version: R004.0.3.783064-Re-20191125-69, Shanghai United Imaging Healthcare Co., Ltd.), this system utilizes a modern 3D Convolutional Neural Network (3D-CNN) optimized for volumetric spatial feature extraction [16].

Vendor B: Integrated with a separate commercial platform (software: lung VCAR, version: AW VolumeShare 7, GE Medical Systems, LLC), this system employs a hybrid architecture combining traditional machine learning morphological filters with deep learning classification layers.

**Figure 1:**
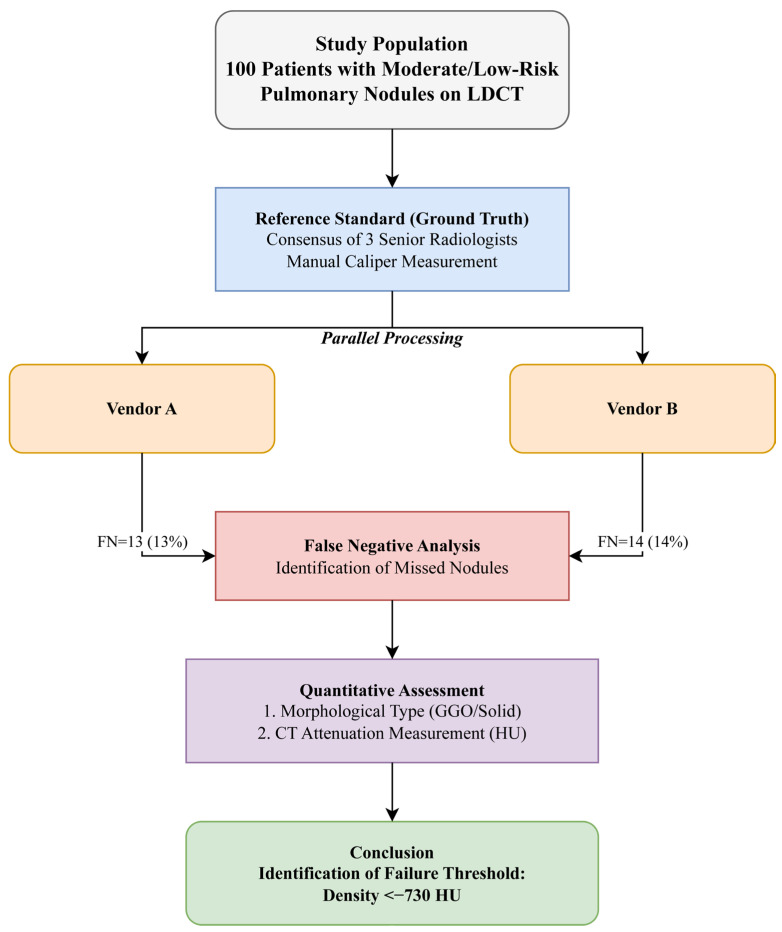
Study flowchart. A diagram illustrating the patient selection process, reference standard establishment, parallel CAD processing, and the analytical steps leading to the identification of the density threshold.

Both systems utilize advanced algorithms for automatic nodule recognition, segmentation, and volumetric measurement. The systems were applied to validated cases to generate automated detection reports. The complete study design and workflow, from patient selection to quantitative analysis, are illustrated in Figure 1.

### 2.5. Data Collection and Measurement

**Nodule Classification:** Nodules were classified by density into solid, sub-solid (mixed density), and pure ground-glass opacities (GGOs).

**Quantitative Analysis:**

(1) *Dimensional Measurement:* For manually detected nodules, the diameter was calculated as the average of the long and short axes ((Length + Width)/2). For CAD-detected nodules, the system automatically generated volume and effective diameter.

(2) *False Negative Analysis:* A false negative (FN) was defined as a nodule present in the reference standard but not marked by the CAD system. To provide a comprehensive performance assessment, false positives (FP) were also recorded, defined as CAD-marked lesions that the expert consensus deemed to be non-nodular structures (e.g., vessels, scars).

(3) *CT Value Measurement:* For all FN nodules, the mean CT attenuation value (in Hounsfield Units, HU) was measured manually by placing a region of interest (ROI) covering at least 70% of the nodule area, avoiding vessels and calcifications. Measurements were meticulously obtained using a 3D spherical tool, strictly maintaining a 1–2 mm distance from the nodule margin to avoid partial volume averaging. The manual ROI measurements demonstrated excellent inter-observer reliability across all data, with an intraclass correlation coefficient (ICC) of 0.92 among the three radiologists. While current manual ROI limits absolute scalability, future models aim to transition towards fully autonomous segmentation formats (such as U-Net architectures) to extract these values directly.

### 2.6. Statistical Analysis

Data analysis was performed using SAS version 9.4 (SAS Institute Inc., Cary, NC, USA). Continuous variables (e.g., CT values, age) are presented as mean ± standard deviation (SD) or median (interquartile range). Categorical variables (e.g., nodule type and detection status) are expressed as frequencies and percentages. Differences between groups were analyzed using the independent samples *t*-test for continuous variables and the Chi-square (χ^2^) test or Fisher’s exact test for categorical variables. A *p*-value of <0.05 was considered statistically significant.

## 3. Results

### 3.1. Distribution of Pulmonary Nodules (Reference Standard)

According to the consensus evaluation by the three senior radiologists, the 100 collected cases in the primary cohort comprised a diverse range of nodule types. There was high inter-observer agreement among the radiologists regarding the classification of nodules (*p* > 0.05), ensuring a robust reference standard (Table 1). The cohort consisted of predominantly solid nodules, followed by GGOs and sub-solid nodules. The external validation cohort (*n* = 50) similarly comprised 30 solid nodules, 17 GGOs, and 3 sub-solid nodules.

### 3.2. False Negative Rates of CAD Systems and Overall Performance

Both CAD systems demonstrated limitations in sensitivity.

**Vendor A** Performance (Primary Cohort): It failed to detect 13 out of 100 nodules, resulting in a false negative rate of 13.0%. The missed nodules were predominantly ground-glass opacities (*n* = 12, 92.31%) with one sub-solid nodule (*n* = 1, 7.69%).

**Vendor B** Performance (Primary Cohort): It failed to detect 14 out of 100 nodules, resulting in a false negative rate of 14.0%. The missed nodules comprised GGOs (*n* = 11, 78.57%), solid nodules (*n* = 2, 14.29%), and a sub-solid nodule (*n* = 1, 7.14%).

**Figure 2:**
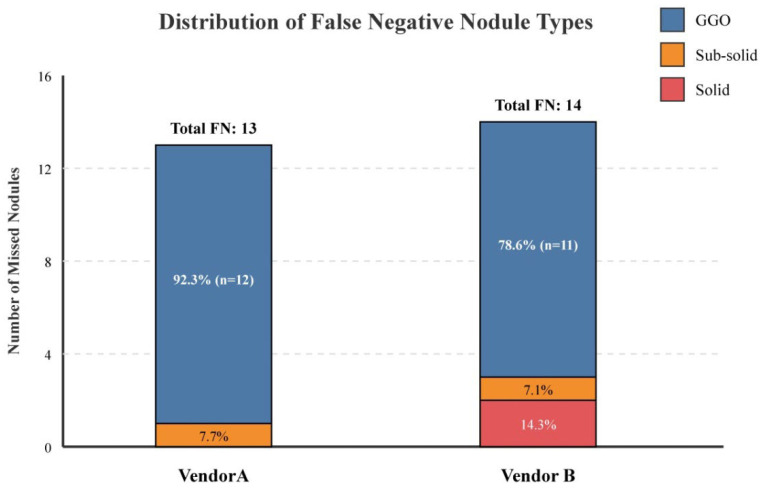
Distribution of false negative nodule types (primary cohort). A bar chart comparing the composition of missed nodules by Vendor A and Vendor B. Ground-glass opacities (blue) constitute the vast majority of false negatives in both systems (92.3% and 78.6%, respectively), while solid nodules (red) were only missed by Vendor B. Data represents findings from the primary cohort (*n* = 100).

There was no statistically significant difference in the overall detection rate between Vendor A (87%) and Vendor B (86%) (*p* > 0.05), suggesting comparable performance between the two vendors. The distribution of false negative nodule types by morphological classification is visually summarized in Figure 2.

External Validation and Confusion Matrix: In the external validation cohort (*n* = 50), Vendor A missed six nodules (12.0%) and Vendor B missed eight nodules (16.0%), corroborating the primary findings. To evaluate the complete diagnostic capability, we computed the full confusion matrices across the total pooled dataset (150 ground-truth nodules). Vendor A recorded 131 true positives (TP), 19 false negatives (FN), and 15 false positives (FP), yielding an overall precision of 89.7% and a recall of 87.3%. Vendor B recorded 128 TP, 22 FN, and 21 FP, resulting in a precision of 85.9% and a recall of 85.3%. Detailed performance metrics are presented in Table 2, and the visual distribution of these confusion matrix metrics is illustrated in Supplementary Figure S1.

### 3.3. Morphological and Anatomical Analysis of False Negatives

**Figure 3:**
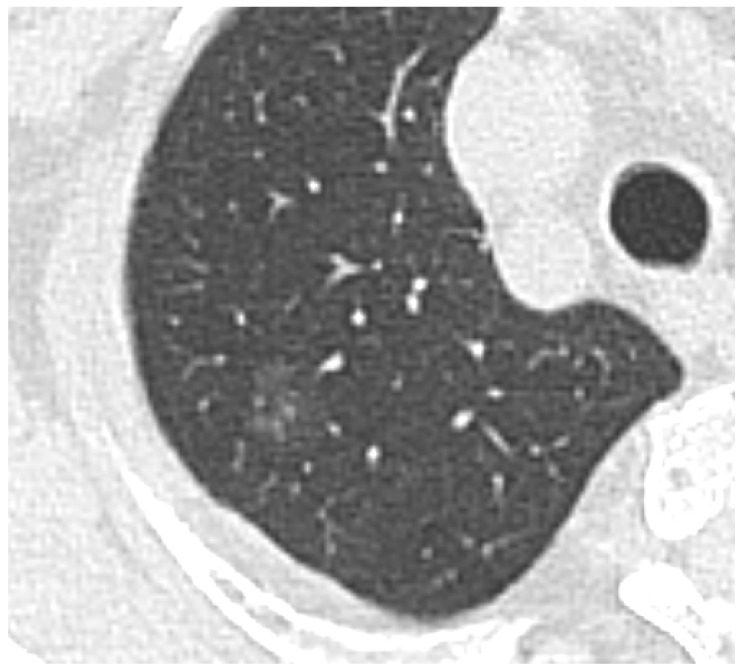
Example of a false negative GGO. The nodule located in the posterior segment of the right upper lobe exhibits low density and distinct vascular convergence (vascular penetration), features that failed to trigger the CAD threshold.

**Figure 4:**
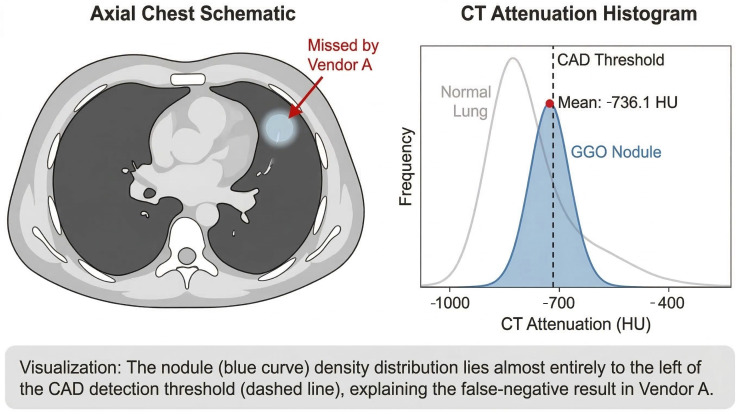
Schematic illustration of a low-density false negative nodule. (**Left**) An axial schematic view showing a smooth GGO in the left upper lobe, which was missed by Vendor A. (**Right**) A density histogram analysis demonstrating the relationship between the nodule’s mean attenuation (−736.1 HU) and the CAD threshold. The nodule’s density distribution (blue curve) lies significantly to the left of the detection threshold (dashed line), explaining the failure of the algorithm to segment this lesion.

**Figure 5:**
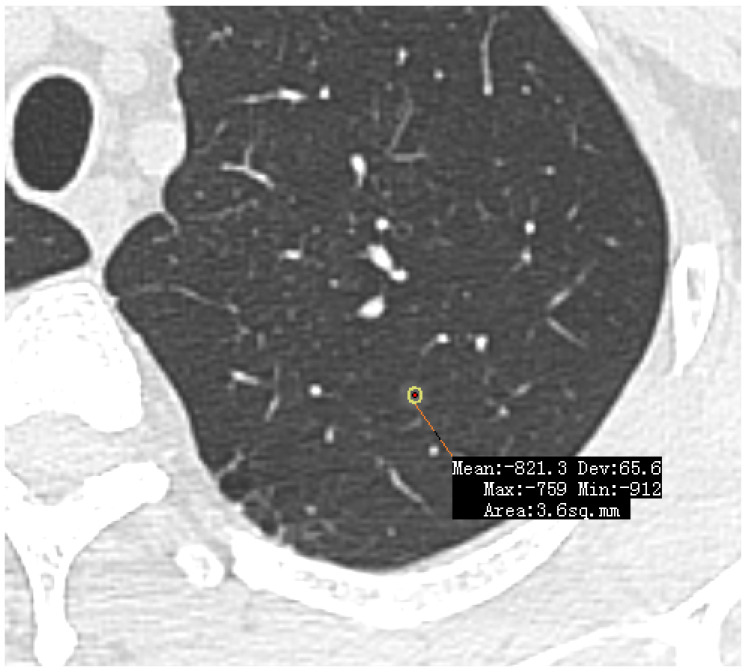
Example of an ultra-low density false negative nodule. An axial HRCT view depicting a faint GGO in the apicoposterior segment of the left upper lobe, missed by both CAD systems. Due to its extremely low CT attenuation (measured at −821 HU), the grayscale difference between the nodule and the surrounding normal lung parenchyma is minimal, making detection highly challenging for standard algorithms. The yellow circle with a red dot represents the manually placed region of interest (ROI) used to measure the mean CT attenuation (−821.3 HU).

Visual inspection of the missed nodules revealed recurring patterns contributing to detection failure:

**(1) Low Density/GGOs:** The vast majority of missed lesions were faint GGOs with indistinct margins. As demonstrated in Figure 3, these nodules exhibit characteristic low density and often present with vascular convergence yet fail to trigger the CAD threshold. Further detailed density histogram analysis illustrating this failure mechanism is presented in Figure 4. Additionally, an extreme visual example of this limitation is depicted in Figure 5, where an ultra-low-density GGO (−821 HU) exhibits minimal grayscale contrast against the surrounding normal lung parenchyma, making it nearly indistinguishable for automated algorithms.

**Figure 6:**
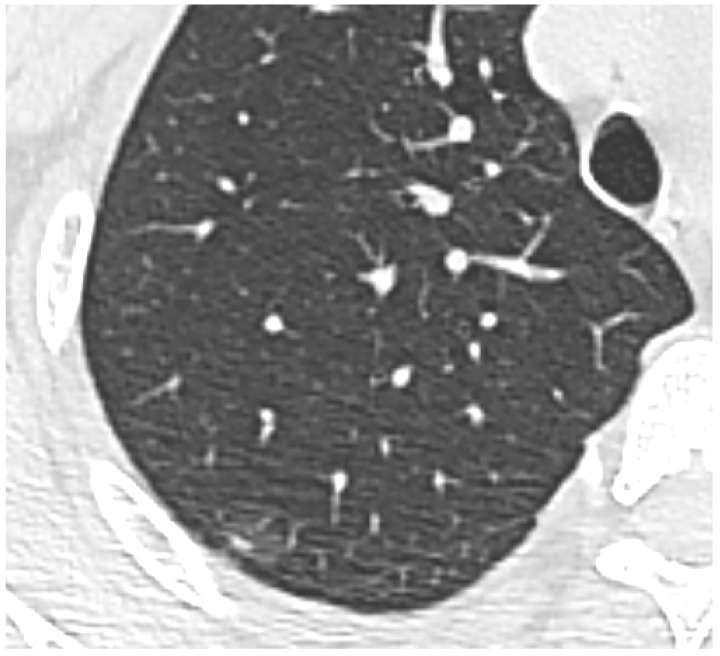
Example of segmentation failure. Magnified HRCT demonstrating a sub-solid nodule in the posterior segment of the right upper lobe. Its irregular margin and immediate proximity to the lung tissue edge (pleura) likely caused the CAD lung segmentation algorithm to exclude the lesion as part of the chest wall.

**(2) Juxta-pleural Location:** Nodules located at the lung periphery, specifically those abutting the pleura, were prone to being excluded by the lung segmentation algorithms of both CAD systems. Figure 6 illustrates a representative case of a sub-solid nodule in the posterior segment of the right upper lobe, which was missed by both systems due to its direct adhesion to the pleural wall, likely causing it to be interpreted as part of the chest wall structure.

**Figure 7:**
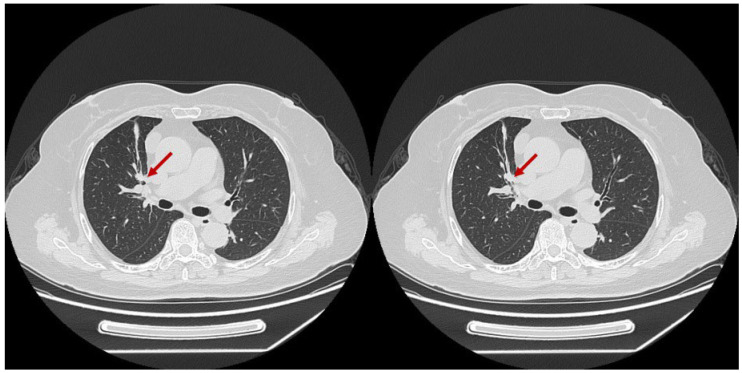
Example of anatomical interference. An intrabronchial solid nodule mimicking normal anatomical structures, leading to a false negative in Vendor B. The red arrows indicate the precise location of the solid nodule within the bronchial lumen.

**(3) Vascular/Bronchial Interference:** Solid nodules located centrally or within the bronchial lumen posed a challenge. Figure 7 depicts a solid nodule located within the bronchus of the anterior segment of the right upper lobe. This lesion was missed by Vendor B, presumably because the algorithm misclassified it as part of the normal bronchial wall or a vascular cross-section due to anatomical interference.

### 3.4. Quantitative CT Value Analysis of Missed GGOs

To determine the density threshold for detection failure, we performed a quantitative analysis of the mean CT attenuation values for the GGOs missed by each system. The results indicate a specific density range where CAD sensitivity drops significantly.

For **Vendor A**, the unrecognized GGOs had a mean CT value of **−737 ± 51.50 HU** (range: −839 to −618 HU) in the primary cohort. For **Vendor B**, the unrecognized GGOs had a mean CT value of **−727 ± 70.07 HU** (range: −839 to −574 HU) in the primary cohort. Crucially, this density threshold was thoroughly corroborated by our external validation cohort, where the mean CT values of missed GGOs were −741 ± 48.2 HU for Vendor A and −733 ± 62.5 HU for Vendor B (Table 3). This reaffirmation highlights the stability of this algorithmic blind spot across different centers and datasets.

This data suggests that nodules with densities approximating −730 HU represent a specific “blind spot” for these commercial CAD algorithms.

## 4. Discussion

The integration of artificial intelligence (AI) into diagnostic radiology has fundamentally altered the landscape of lung cancer screening. While computer-aided detection (CAD) systems serve as powerful adjuncts for nodule detection, volumetry, and density analysis [17], our study underscores a critical caveat: they are not infallible. By comparing two widely used commercial CAD systems against a robust radiologist consensus standard, we identified a consistent false negative rate of 13–14%, with a predominant failure in detecting ground-glass opacities (GGOs). This finding is pivotal because persistent GGOs have a significantly higher malignancy rate (up to 34%) compared to solid nodules (7%) [18], often representing pre-invasive or minimally invasive adenocarcinomas (AIS/MIA) that offer excellent prognosis if resected early.

### 4.1. Performance Benchmarking and False Negative Rates

Our calculated sensitivity for Vendor A (87%) and Vendor B (86%) aligns with the current literature, which reports CAD sensitivities ranging from 38% to 98% depending on the algorithm generation and nodule types [19]. Specifically, Katase et al. reported a sensitivity of 98% using a newer deep-learning model [20], while Murchison et al. achieved 95.9% sensitivity with a deep learning CAD system [7]. In contrast, Kozuka et al. reported a sensitivity as low as 38% on 1-mm-thick images, highlighting performance variation under specific conditions [21]. Our results sit in the median range, likely reflecting the real-world performance of standard commercial packages currently installed in many hospitals, which may not yet fully leverage state-of-the-art deep learning architectures for all nodule types. The inclusion of complete confusion matrices further demonstrates that while recall remains stable, the systems also produce a modest rate of false positives, illustrating the classic precision–recall trade-off inherent in AI classification.

### 4.2. The “Density Blind Spot”: Quantitative Thresholds for Failure

The most significant contribution of this study is the quantitative identification of a “density blind spot.” We found that both CAD systems consistently failed to identify GGOs with mean CT attenuation values clustering around −730 HU (Vendor A: −737 ± 51.50 HU; Vendor B: −727 ± 70.07 HU). This threshold is clinically actionable. It suggests that when the density of a GGO drops below approximately −700 HU, the contrast-to-noise ratio (CNR) between the lesion and the surrounding aerated lung parenchyma becomes insufficient for the segmentation algorithms to distinguish pathology from background noise, a fundamental limitation acknowledged in recent AI model evaluations [22].

Recent studies corroborate this “low-contrast failure” phenomenon. For instance, research has demonstrated that AI models struggle with low-density sub-solid nodules due to feature overlap with normal alveolar structures, leading to significant detection gaps in clinical screening [23]. Unlike solid nodules, which present strong gradients, faint GGOs lack distinct boundaries, causing traditional morphological filters and even some convolutional neural networks (CNNs) to suppress them as noise [24]. This persistent challenge underscores a specific area where human visual perception—trained to recognize subtle textural changes—still outperforms automated detection, as highlighted in contemporary reviews of deep learning applications in pulmonary imaging [25].

### 4.3. Anatomical and Structural Interference

Beyond density, anatomical location proved to be a major determinant of detection failure. Our analysis of the false negative cases identified two primary structural challenges:

(1) Pleural Adhesion: Nodules abutting the pleura were frequently missed. This is likely due to the initial lung segmentation step in CAD pipelines. A key challenge in automated segmentation is to include juxta-pleural nodules, as they are affixed to the chest wall and share a similar intensity profile, causing most methods to fail to include them in the segmented lung parenchyma [26]. If the algorithm aggressively smooths the lung boundary to remove the chest wall, juxta-pleural nodules may be inadvertently “cropped out.”

(2) Vascular/Bronchial Mimicry: Solid nodules located centrally or endobronchially (Figure 3) mimic the cross-sectional appearance of vessels or bronchial walls. In our study, Vendor B missed two solid nodules for this reason. This aligns with documented performance limitations, where common reasons for CAD missing nodules include their proximity to vessels, which remains a significant source of false negatives in automated systems [27].

### 4.4. Clinical Implications

These findings translate into specific recommendations for clinical practice:

1. Targeted Manual Review: Radiologists should not blindly trust a “negative” CAD report. A targeted manual review is essential for two specific regions: the lung periphery (for juxta-pleural nodules) and the central hilar regions (for endobronchial lesions).

2. Sensitivity to “Faint” Lesions: When reviewing LDCT scans, radiologists must maintain a high index of suspicion for extremely faint opacities (<−700 HU), as these are the precise lesions AI is most likely to miss.

3. Contextualizing Indolent Disease: Crucially, detecting every minute lesion is not unequivocally beneficial. As emphasized by the Fleischner Society Guidelines (2017) [12] and prospective studies by Kakinuma et al. [13], GGOs measuring 5 mm or less frequently exhibit negligible volumetric progression. The unwarranted identification of such indolent nodules may precipitate severe psychological distress for the patient and impose an unjustifiable economic burden through excessive surveillance. Therefore, while CAD false negatives represent a technical limitation, recognizing this blind spot also prevents aggressive overtreatment of lesions that may not be deleterious to the patient’s long-term health.

4. Algorithm Selection: Hospitals procuring AI software should specifically inquire about the vendor’s validation data for sub-solid and GGO nodules, prioritizing systems that have been trained on datasets enriched with low-density lesions.

### 4.5. Limitations

This study has several limitations. First, the primary sample size of 100 cases and 50 external cases is relatively small, although sufficient to identify statistically significant trends in false negative characteristics. Second, as a retrospective study, selection bias cannot be entirely excluded. Third, while we utilized “consensus expert reading” as the reference standard, pathological confirmation was not available for all cases, as many low-risk nodules are managed with surveillance rather than immediate resection. Future studies should employ larger, multi-center datasets with pathological correlation to validate these density thresholds further. Additionally, while our manual ROI measurements demonstrated excellent inter-observer reliability, the manual nature of this approach limits absolute scalability. Future large-scale screening protocols will benefit from transitioning to fully automated 3D segmentation architectures (such as U-Net) to extract precise density metrics, thereby minimizing subjective bias and improving workflow efficiency.

## 5. Conclusions

In conclusion, while commercial CAD systems are invaluable tools for lung nodule screening, they exhibit distinct limitations in detecting moderate and low-risk nodules. Specifically, they demonstrate a high false negative rate for ground-glass opacities with mean CT values approximating −730 HU and for nodules located adjacent to the pleura or within bronchial structures. The “density blind spot” identified in this study serves as a critical alert for radiologists: CAD is not omnipotent. To prevent the missed diagnosis of potentially malignant early-stage lesions, a hybrid workflow combining AI detection with targeted expert manual verification—focusing on low-density and anatomically complex areas—is essential, provided that interventions are carefully weighed against the risks of overdiagnosis as outlined by contemporary guidelines.

## Figures and Tables

**Table 1:** Distribution of moderate and low-risk pulmonary nodules in the primary and external validation cohorts.

Nodule Type	Primary Cohort (*n* = 100)	External Cohort (*n* = 50)	Percentage (Pooled, *n* = 150)	Typical Features
Solid Nodule	66	30	64.0%	Soft tissue density, obscuring underlying vessels
Ground-Glass Opacity (GGO)	29	17	30.7%	Hazy increased opacity, preservation of bronchial/vascular margins
Sub-Solid Nodule	5	3	5.3%	Contains both ground-glass and solid components

**Table 2:** Complete confusion matrix and performance metrics for CAD systems (pooled cohort, *n* = 150 ground-truth nodules).

System	True Positives (TP)	False Negatives (FN)	False Positives (FP)	Precision (%)	Recall/Sensitivity (%)	F1-Score
Vendor A	131	19	15	89.7%	87.3%	0.885
Vendor B	128	22	21	85.9%	85.3%	0.856

**Table 3:** Quantitative CT attenuation analysis of ground-glass nodules missed by CAD systems.

System	Primary Cohort (*n* = 100)			External Validation Cohort (*n* = 50)		
	Missed GGOs (n)	Mean CT Value (HU) ± SD	Range (Min~Max HU)	Missed GGOs (n)	Mean CT Value (HU) ± SD	Range (Min~Max HU)
Vendor A	12	−737 ± 51.50	−839~−618	5	−741 ± 48.20	−825~−672
Vendor B	11	−727 ± 70.07	−839~−574	6	−733 ± 62.50	−842~−645

## Data Availability

The original contributions presented in this study are included in the article. Further inquiries can be directed to the corresponding author.

## Supplementary Materials

The following supporting information can be downloaded at: https://www.mdpi.com/article/10.3390/diagnostics16071014/s1, Figure S1: Distribution of confusion matrix metrics for CAD systems across the pooled cohort.

## Abbreviations

CAD	Computer-Aided Detection
CNR	Contrast-to-Noise Ratio
FN	False Negative
GGO	Ground-Glass Opacity
HU	Hounsfield Units
LDCT	Low-Dose Computed Tomography
MSCT	Multi-Slice Computed Tomography
PACS	Picture Archiving and Communication System
ROI	Region of Interest
SD	Standard Deviation

## References

[1] Bray F., Laversanne M., Sung H., Ferlay J., Siegel R.L., Soerjomataram I., Jemal A. (2024). Global cancer statistics 2022: GLOBOCAN estimates of incidence and mortality worldwide for 36 cancers in 185 countries. CA Cancer J. Clin..

[2] Bai J., Fu F., Sun W., Deng C., Ma Z., Wang S., Deng L., Zhang Y., Chen H. (2023). Prognostic effect of ground-glass opacity in subcentimeter invasive lung adenocarcinoma. J. Thorac. Dis..

[3] Kim H.Y., Shim Y.M., Lee K.S., Han J., Yi C.A., Kim Y.K. (2007). Persistent pulmonary nodular ground-glass opacity at thin-section CT: Histopathologic comparisons. Radiology.

[4] Henschke C.I., Yankelevitz D.F., Mirtcheva R., McGuinness G., McCauley D., Miettinen O.S. (2002). CT screening for lung cancer: Frequency and significance of part-solid and nonsolid nodules. AJR Am. J. Roentgenol..

[5] Zhu M., Xu Y., Huang J., Yao Y., Tosi D., Koike T., Villamizar N.R., Wang Z., Mao F., Luo Q. (2024). Sublobar resection for lung adenocarcinoma less than 2 cm containing solid or micropapillary components radiologically presented as consolidation-to-tumor ratio (CTR) ≤0.25 [ground-glass opacity (GGO)]. Transl. Lung Cancer Res..

[6] Peloschek P., Sailer J., Weber M., Herold C.J., Prokop M., Schaefer-Prokop C. (2007). Pulmonary nodules: Sensitivity of maximum intensity projection versus that of volume rendering of 3D multidetector CT data. Radiology.

[7] Murchison J.T., Ritchie G., Senyszak D., Nijwening J.H., van Veenendaal G., Wakkie J., van Beek E.J.R. (2022). Validation of a deep learning computer aided system for CT based lung nodule detection, classification, and growth rate estimation in a routine clinical population. PLoS ONE.

[8] Grenier P.A., Brun A.L., Mellot F. (2022). The Potential Role of Artificial Intelligence in Lung Cancer Screening Using Low-Dose Computed Tomography. Diagnostics.

[9] Zarinshenas R., Amini A., Mambetsariev I., Abuali T., Fricke J., Ladbury C., Salgia R. (2023). Assessment of Barriers and Challenges to Screening, Diagnosis, and Biomarker Testing in Early-Stage Lung Cancer. Cancers.

[10] Kang S., Kim T.H., Shin J.M., Han K., Kim J.Y., Min B., Park C.H. (2020). Optimization of a chest computed tomography protocol for detecting pure ground glass opacity nodules: A feasibility study with a computer-assisted detection system and a lung cancer screening phantom. PLoS ONE.

[11] Park S., Lee S.M., Kim W., Park H., Jung K.H., Do K.H., Seo J.B. (2021). Computer-aided Detection of Subsolid Nodules at Chest CT: Improved Performance with Deep Learning-based CT Section Thickness Reduction. Radiology.

[12] MacMahon H., Naidich D.P., Goo J.M., Lee K.S., Leung A.N.C., Mayo J.R., Mehta A.C., Ohno Y., Powell C.A., Prokop M. (2017). Guidelines for Management of Incidental Pulmonary Nodules Detected on CT Images: From the Fleischner Society 2017. Radiology.

[13] Kakinuma R., Noguchi M., Ashizawa K., Kuriyama K., Maeshima A.M., Koizumi N., Kondo T., Matsuguma H., Nitta N., Ohmatsu H. (2016). Natural History of Pulmonary Subsolid Nodules: A Prospective Multicenter Study. J. Thorac. Oncol. Off. Publ. Int. Assoc. Study Lung Cancer.

[14] Bossuyt P.M., Reitsma J.B., Bruns D.E., Gatsonis C.A., Glasziou P.P., Irwig L., Lijmer J.G., Moher D., Rennie D., de Vet H.C. (2015). STARD 2015: An updated list of essential items for reporting diagnostic accuracy studies. BMJ.

[15] Zhou Q., Fan Y., Wang Y., Qiao Y., Wang G., Huang Y., Wang X., Wu N., Zhang G., Zheng X. (2016). [China National Guideline of Classification, Diagnosis and Treatment for Lung Nodules (2016 Version)]. Zhongguo Fei Ai Za Zhi = Chin. J. Lung Cancer.

[16] Guo H., Wu J., Xie Z., Tham I.W.K., Zhou L., Yan J. (2022). Investigation of small lung lesion detection for lung cancer screening in low dose FDG PET imaging by deep neural networks. Front. Public Health.

[17] Paramasamy J., Mandal S., Blomjous M., Mulders T., Bos D., Aerts J., Vanapalli P., Challa V., Sathyamurthy S., Devi R. (2025). Validation of a commercially available CAD-system for lung nodule detection and characterization using CT-scans. Eur. Radiol..

[18] Austin J.H., Garg K., Aberle D., Yankelevitz D., Kuriyama K., Lee H.J., Brambilla E., Travis W.D. (2013). Radiologic implications of the 2011 classification of adenocarcinoma of the lung. Radiology.

[19] Gao C., Wu L., Wu W., Huang Y., Wang X., Sun Z., Xu M., Gao C. (2025). Deep learning in pulmonary nodule detection and segmentation: A systematic review. Eur. Radiol..

[20] Katase S., Ichinose A., Hayashi M., Watanabe M., Chin K., Takeshita Y., Shiga H., Tateishi H., Onozawa S., Shirakawa Y. (2022). Development and performance evaluation of a deep learning lung nodule detection system. BMC Med. Imaging.

[21] Kozuka T., Matsukubo Y., Kadoba T., Oda T., Suzuki A., Hyodo T., Im S., Kaida H., Yagyu Y., Tsurusaki M. (2020). Efficiency of a computer-aided diagnosis (CAD) system with deep learning in detection of pulmonary nodules on 1-mm-thick images of computed tomography. Jpn. J. Radiol..

[22] Shah H.P., Naqvi A.S., Rajput P., Ambra H., Venkatesh H., Saleem J., Saravanan S., Wanjari M., Mittal G. (2025). Artificial intelligence-based deep learning algorithms for ground-glass opacity nodule detection: A review. Narra J.

[23] Wu H., Zhang Y., Hu H., Li Y., Shen X., Liu Q., Wang S., Chen H. (2021). Ground glass opacity featured lung adenocarcinoma in teenagers. J. Cancer Res. Clin. Oncol.

[24] Ding Y., He C., Zhao X., Xue S., Tang J. (2022). Adding predictive and diagnostic values of pulmonary ground-glass nodules on lung cancer via novel non-invasive tests. Front. Med..

[25] Ye W., Gu W., Guo X., Yi P., Meng Y., Han F., Yu L., Chen Y., Zhang G., Wang X. (2019). Detection of pulmonary ground-glass opacity based on deep learning computer artificial intelligence. Biomed. Eng. Online.

[26] Rikhari H., Baidya Kayal E., Ganguly S., Sasi A., Sharma S., Dheeksha D.S., Saini M., Rangarajan K., Bakhshi S., Kandasamy D. (2024). Fully automatic deep learning-based lung parenchyma segmentation and boundary correction in thoracic CT scans. Int. J. Comput. Assist. Radiol. Surg..

[27] Bhure U., Cieciera M., Lehnick D., Del Sol Pérez Lago M., Grünig H., Lima T., Roos J.E., Strobel K. (2023). Incorporation of CAD (computer-aided detection) with thin-slice lung CT in routine 18F-FDG PET/CT imaging read-out protocol for detection of lung nodules. Eur. J. Hybrid Imaging.

