# Supplementary material for: Identifying a Critical Blind Spot: How Commercial AI (CAD) Systems Fail to Detect Faint Ground-Glass Opacities at −730 HU on Low-Dose CT
Source: Diagnostics (Basel). 2026 Mar 27;16(7):1014. doi: 10.3390/diagnostics16071014 (PMC13072957; doi:10.3390/diagnostics16071014)
Supplement: Supplementary file 1 [file diagnostics-16-01014-s001.zip › diagnostics-4152147-supplementary.pdf]

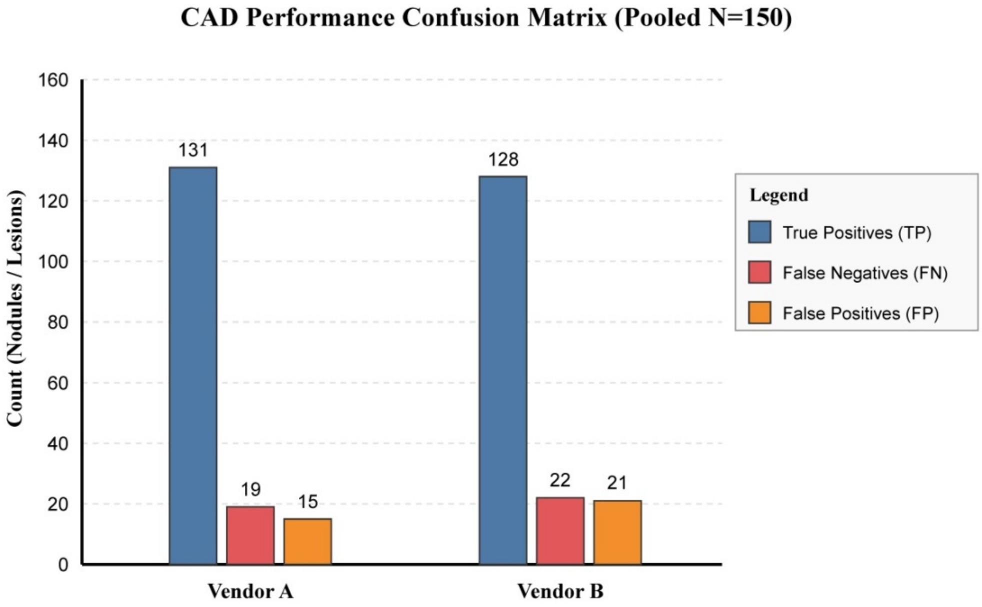

Supplementary Figure S1. CAD performance: confusion matrix breakdown. A bar chart illustrating the complete diagnostic performance of Vendor A and Vendor B across the pooled dataset (n = 150 ground-truth nodules). The chart visualizes the distribution of true positives (TP), false negatives (FN), and false positives (FP) for each system, demonstrating the precision–recall trade-off inherent in these AI algorithms.
